# Pennsylvania Hospital—Surgical Wards—Service of Dr. Norris

**Published:** 1842-04-09

**Authors:** E. Hartshorne

**Affiliations:** Resident Surgeon


### CLINICAL REPORTS.

### *Pennsylvania Hospital—Surgical Wards—Service of Dr. Norris.*

By Dr. E. Hartshorne, Resident Surgeon.

*Compound fracture of the leg.*—J. W., æt. 28, temperate, not very robust, and affected with asthma, was admitted Nov. 19th, with a compound fracture of the left tibia and fibula rather below their middle. The fracture was transverse, and had been received twenty-two days previously at sea. According to his statement, the injury resulted from the blow of a heavy chain-cable violently thrown against the part from the windward side of the vessel in a sudden lurch of the latter. The tibia was driven through the integuments anteriorly, so as to protrude at least two inches, while the neighbouring soft parts were severely contused. The displaced fragments were pretty well reduced and fixed in short splints by the master of the ship. This apparatus unfortunately neither prevented some displacement nor secured rest; the man's sufferings, therefore, were much increased by the continued jarring to which he was exposed by the unmitigated roughness of the weather during the remainder of the voyage, as well as by repeated paroxysms of asthma, always agravated by the confinement in a close cabin.

When brought into the wards he was emaciated, pale and feeble. The injured extremity was still bound up in splints about ten inches long, and the wound was covered with a muslin rag moistened with whiskey. The removal of this rude dressing exposed to view nearly an inch of dark coloured bone, which proved to be the anterior angle of the upper fragment protruding in front. No inflammation existed at that time in the leg, and little or no discharge flowed from the granulating surface. Although considerable motion and great tenderness still remained in the seat of fracture, it was too late to reduce the displaced fragment. Its uncovered portion, therefore, was excised, and the remaining ulcer dressed twice a day with a small poultice; while the leg was placed at rest in a fracture box as usual, with the heel carefully supported. The patient was allowed a good diet, and half a pint of porter daily, under which he rapidly gained flesh and improved in strength. During his confinement to bed in the wards, the man was subjected to frequent paroxysms of asthma, some of which were quite severe. They were generally combatted, when requisite, by nervous stimulants, nauseants, &c., internally, and local depletion and counter irritation, externally. In about sixty days a small lamen of bone exfoliated; after which the ulcer which had become very circumscribed, soon cicatrized. Consolidation, which was at this time rapidly advancing, at the end of seventy days appeared to be complete. On the seventy-fourth day the paste-board splints were applied, and the patient permitted to walk with crutches. He was discharged Feb. 28, with a perfectly good limb.

